# Are Thiols Useful Biomarkers for Coronary Collateral Circulation in Patients with Stable Coronary Artery Disease?

**DOI:** 10.3390/jcm12196361

**Published:** 2023-10-04

**Authors:** Yasemin Doğan, Yücel Yilmaz, Saban Kelesoğlu, Bekir Calapkorur, Salim Neşelioglu, Özcan Erel, Nihat Kalay

**Affiliations:** 1 Department of Cardiology, Kayseri City Training and Research Hospital, University of Health Sciences, Kayseri 38080, Turkey; dryyilmaz@hotmail.com (Y.Y.); drcalapkorur@yahoo.com (B.C.); 2 Department of Cardiology, Erciyes University Faculty of Medicine, Kayseri 38039, Turkey; dr.s.k@hotmail.com (S.K.); nihatkalay@hotmail.com (N.K.); 3 Department of Biochemistry, Yildirim Beyazit University, Ankara 06800, Turkey; salim.neselioglu@gmail.com (S.N.); erelozcan@gmail.com (Ö.E.)

**Keywords:** coronary collateral circulation, stable coronary artery disease, thiol

## Abstract

Our aim was to investigate the relationship between thiol, which is the main component of the antioxidant system, and coronary collateral circulation (CCC). Our patients consisted of people with stable coronary artery disease (sCAD) and total occlusion in at least one vessel (*n* = 249). We divided the patients into two groups, good and poor, according to their CCC degree. We determined that DM, total thiol, and disulfide are independent predictors of poor CCC in multivariate logistic regression analysis (OR: 1.012, 95% CI: 1.008–1.017, *p* < 0.001; OR: 1.022, 95% CI: 1.000–1.044, *p* = 0.044; OR: 2.671, 95% CI: 1.238–5.761, *p* = 0.012, respectively). The ROC analysis showed a cut-off value of 328.7 for native thiol regarding the prediction of poor CCC, with 67.4% specificity and 78% sensitivity. For disulfide, it revealed a cut-off value of 15.1 regarding the prediction of poor CCC, with 57.9% specificity and 69.5% sensitivity. In this study, we detected that the patients with sCAD who developed poor CCC had lower levels of native thiol, total thiol, and disulfide compared to those with good CCC. The most interesting finding of our study is that CCC formation is an effective predictor of the antioxidant cascade rather than the inflammation cascade in sCAD patients.

## 1. Introduction

The small blood vessel channels linking coronary vessels to each other (between different coronary arteries or between different segments of the same coronary artery) are implicated in something called coronary collateral circulation (CCC). As a result of stimulation via chronic and/or recurrent myocardial ischemia, inactive collateral vessels open and support blood flow depending on the duration and severity of ischemia [1]. CCC has the potential to determine the myocardial infarction size and clinical sequelae of CAD [2]. In addition, coronary artery patients with good CCC have been shown to have a myocardium that is better protected from ischemia, better left ventricular functions, fewer subsequent cardiac events, and lower mortality compared to patients with poor CCC [3,4]. Although myocardial ischemia is a major factor in CCC growth, the reason why different CCC connections develop between individuals with similar ischemia-induced heart disease remains unclear. Drugs (nitrate, calcium channel blockers, statin), endothelial dysfunction and the coronary vasomotor tone are important in CCC formation. In addition, it is known that high levels of sensitive C-reactive protein (CRP), lipoprotein (a), adhesion molecules (e.g., vascular adhesion molecule-1), intercellular adhesion molecule-1, various serum markers (e.g., E-selectin and tumor necrosis factor-a), and antioxidant capacity are associated with the degree of CCC development [5,6,7,8,9,10,11,12,13].

Reactive oxygen radicals, which are compounds with high activity, and oxidants like reactive oxygen species (ROS), which are products of aerobic cellular metabolism, occur in the cellular environment [14,15,16]. ROS can oxidatively modify or damage lipids, proteins, and DNA, which may have detrimental consequences for vascular function and structure [17,18]. Such damage plays a significant role in the emergence of chronic diseases such as vascular diseases, neurodegeneration, immunological disorders, aging, and cancer [19]. Thiol groups comprise an antioxidant cascade that is noteworthy regarding the elimination of ROS [20,21]. The antioxidant components of this group that ensure homeostasis are natural thiol, disulfide and total thiol. Thiols are organic compounds containing the sulfhydryl (-SH) group that react with reactive oxidant molecules and neutralize them. Their main function is to prevent any oxidative stress within cells and organisms [21,22]. Upon causing an antioxidation reaction, thiols cause the formation of reversible disulfide bonds. Adverse changes in the thiol/disulfide balance cause ROS formation.

Oxidative stress and inflammation are two systems that interact in parallel with each other, and both have impacts at every stage of CAD. Previous studies have suggested that there is an association between the platelet lymphocyte ratio (PLR) and neutrophil lymphocyte ratio (NLR) and CCC formation in patients with stable angina pectoris [23,24]. Although the relevant literature covers a few studies showing the relationship between the antioxidant system and CCC, we still do not know the relationship between thiol and CCC. Ultimately, our aim in this study was to investigate the relationship between thiol, which is the main component of the antioxidant system, and CCC. 

## 2. Materials and Method

This was a single-center and prospective study conducted with patients presented to Kayseri City Hospital’s Cardiology Clinic between March 2019 and November 2020. In total, 249 patients were included in our study. The patients were those diagnosed with sCAD according to the criteria of the European Society of Cardiology, those who had underwent coronary angiography (due to the results of non-invasive stress tests suggesting typical chest pain or myocardial ischemia [positive stress test result and/or ischemia suggested by myocardial perfusion scintigraphy], and those detected to have at least one total occlusion of the main coronary vessel [25]. The medical histories of all participants were recorded. All patients were subjected to routine physical examinations and transthoracic echocardiography.

We excluded patients who had previously undergone coronary artery bypass grafting (CABG) or percutaneous coronary intervention (PCI), and those who had history of an acute coronary syndrome in the previous 3 months, hematological disease, malignancy, chronic renal failure or liver disease, and ongoing infection or chronic inflammatory disease. Those receiving autoimmune disease or vitamin supplements were also not included in the study.

All patients provided their written informed consent before participating in the study. The research protocol of the present research, which is in line with the Declaration of Helsinki, was approved by the local research ethics committee.

For laboratory analysis, we took blood samples from all the patients before coronary angiography, between 08:00–10:00 in the morning following a 12 h fasting period. We transferred the antecubital venous blood samples into tripotassium EDTA-based anticoagulated tubes. We used the blood samples to measure essential blood variables (e.g., a comprehensive metabolic panel and complete blood count) and thiol levels. We ran all routine biochemical tests using an autoanalyzer (Olympus AU2700 analyzer, Beckman Coulter, Tokyo, Japan). For hematological parameters, the samples were stored at 4 °C and analyzed with a Mindray BC-6800 auto analyzer (Mindray Bio-Medical Electronics, Nanshan, Shenzhen, China) within 30 min of sampling. We calculated the NLR by dividing the number of neutrophils by the number of lymphocytes. We found the PLR by dividing the platelet count by the number of lymphocytes.

We measured the levels of thiol groups as described by Erel et al. [26]. Thiol samples were centrifuged at 1500× *g* for 10 min. We stored plasma at −80 °C and processed all samples simultaneously. We detected the natural thiol and total thiol levels spectrophotometrically. First, we measured the natural thiol levels after reaction with 5,5′-dithiobis-2-nitrobenzoic acid (DTNB) without any treatment. Secondly, to measure the total thiol levels, we reduced the dynamic disulfide bonds in the serum samples using sodium borohydride (NaBH_4_) to form free functional thiol groups. Then, we used formaldehyde to eradicate unused NaBH_4_ and measured the total thiol groups, including both reduced and natural ones, following reaction with DTNB. We calculated the number of dynamic disulfide bonds by determining half the difference between the natural thiol and total thiol. The percent coefficient variation (%CV) was 4 ($\bar{X}$ = 29.12 and σX = 1.2) for high levels, 5 ($\bar{X}$ = 16.03 and σX = 0.79) for medium levels and 13 ($\bar{X}$ = 7.15 and σX = 0.98) for low levels. We performed transthoracic echocardiography for each patient before coronary angiography. We made all measurements using a commercially available machine (Vivid 5, GE Medical System, Horten, Norway) with a 3.5 MHz transducer. We made 2D echocardiographic measurements to evaluate the left ventricular ejection fraction and valve pathologies. In the apical 4-chamber view, we utilized Simpson’s method and color doppler echocardiography to assess the ejection fraction and valve pathologies, respectively. We performed selective coronary angiography (CAG) according to the Judkins technique using a 6 or 7 French (F) catheter and a right or left femoral interventional approach. Iohexol (Omnipaque^®^ 350 mg/mL) and lopromide (Ultravist-370^®^) were the opaque agents we used. We visualized the coronary arteries in all patients with cranial and caudal tilt in the left and right oblique planes.

The coronary collateral circulation was graded using the Rentrop classification: grade 0 = no filling of any collateral vessel; grade 1 = filling of side branches of the artery to be the epicardial segment; grade 2 = partial filling of the epicardial artery by collateral vessels; and grade 3 = complete filling of the epicardial artery by a collateral vessel [27]. We accepted Rentrop grade 0 as no development of CCC (Group 2). Also, we considered Rentrop grades 0–1 to be poor CCC development and Rentrop grades 2–3 to be good CCC development (Group 1). We considered all coronary lesions causing 50% stenosis in vessels greater than 1.5 mm in diameter for the calculation of the SYNTAX score. The SYNTAX score was calculated based on the latest online version of the software available on the website [28]. Two experienced cardiologists evaluated the CAG images of the patients.

We ran the statistical analyses using SPSS 21.0 (SPSS Inc., Chicago, IL, USA) software for Windows. We checked the normality of the distribution of the quantitative data using the Shapiro–Wilk test. We presented descriptive data as mean ± standard deviation and median (interquartile range, IQR), depending on the normality of the distribution. We displayed non-normally distributed data as median and interquartile range. We utilized the independent samples *T*-test to compare the normally distributed quantitative variables, while the Mann–Whitney U test was used to compare the non-normally distributed data. We run the Chi-square test to compare categorical variables. The impacts of different variables on CCC formation were calculated using univariate analysis. For multivariate regression analysis, we included parameters with *p* < 0.10 in univariate analysis in the model. The cut-off levels of thiol and NLR in predicting CCC formation were determined using the receiver operating characteristic curve analysis (ROC). In all statistical analyses, we considered *p* values less than 0.05 to be statistically significant.

## 3. Results

We noted a total of 249 patients with sCAD and those with the complete occlusion of at least a coronary artery on CAG. Table 1 and Table 2 present the baseline clinical, demographic characteristics and laboratory findings. We divided the patients into two groups according to the Rentrop classification (good CCC (Rentrop 1–2); poor CCC (Rentrop 0–1)). Accordingly, 49 patients had poor collateral development (Rentrop 0–1), while 200 had good collateral formation (Rentrop 2–3). We could not find a significant difference between the groups according to hypertension, dyslipidemia, gender, smoking status, BMI, systolic and blood pressure, heart rate, LVEF, drug use (Aspirin, Β-blocker, Angiotensin–aldosterone antagonists, Statin, Clopidogrel). We discovered more prevalent older age, diabetes mellitus (DM) and more elevated hsCRP levels in the group of patients with poor CCC (*p* = 0.015, *p* = 0.004 and *p* = 0.012, respectively).

Table 3 shows the angiographic data of the patients. There was no statistical difference between the two groups in terms of the location of the affected coronary arteries, the number of vessels and the SYNTAX score.

In addition, we sought the role of various risk factors in the occurrence of CCC using multivariate analysis. We performed multivariate logistic regression analysis with values shown to be associated with poor CCC formation in univariate analysis (DM, age, platelet, hsCRP, NLR, total thiol, and disulfide). The regression analysis revealed that the total thiol, disulfide, and DM were independent predictors of poor CCC (OR: 1.012, 95% CI: 1.008–1.017 *p* < 0.001; OR: 1.022, 95% CI: 1.000–1.044 *p* = 0.044; OR: 2.671, 95% CI: 1.238–5.761 *p* = 0.012, respectively) (Table 4).

**Figure 1:**
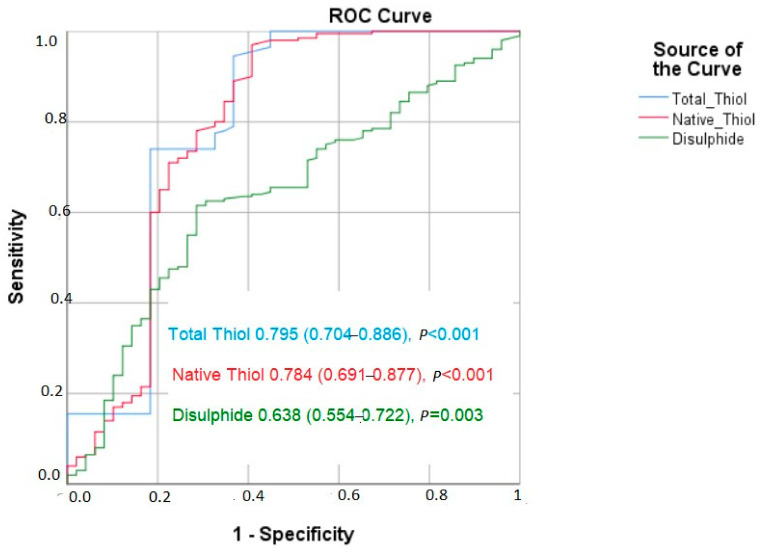
The ROC curve of thiols and coronary collateral circulation.

The results of the ROC analysis revealed that native thiol had a cut-off value of 328.7 (area under the curve (AUC) = 0.784 (95% CI: 0.691–0.877, *p* < 0.001)) regarding the prediction of poor CCC, with 78% sensitivity and 67.4% specificity. For the total thiol, a cut-off value of 355.9 predicted poor CCC, with 77.5% sensitivity and 67.3% specificity (AUC = 0.795 (95% CI: 0.704–0.886, *p* < 0.001). In addition, the results showed a sensitivity of 69.5% and specificity of 57.9% at the cut-off value of 15.1 for disulfide, indicating poor CCC (AUC = 0.638 [0.554–0.722]) (Figure 1).

## 4. Discussion

In this study, we found that sCAD patients who developed poor CCC had lower levels of native thiol, total thiol, and disulfide compared to those with good CCC. In addition, the likelihood of DM was significantly higher in patients with poor CCC. The most striking finding of our study was that the antioxidant cascade was an effective predictor of CCC formation in patients with sCAD, rather than the inflammation cascade.

CCC, also considered a natural bypass, provides an important alternative blood supply when the natural vein cannot do so. Only approximately 25–35% of those with chronic total occlusion (CTO) have functional CCC to prevent myocardial ischemia [30]. However, in recent CCC studies conducted in our country (without evaluating whether it affects coronary ischemia), good CCC development rates have been found to be more than 60% [31,32]. In fact, in two studies conducted by Kelesoglu et al. [33,34], this rate approached 80%. In our study, we found the good CCC development rate to be approximately 80%. We have seen that this rate is higher when compared to the literature. We can speculate that the reason for the higher number of patients with good CCC in our study is that our patients were exposed to CTO for a longer period of time, together with genetic, ethnic and regional variables.

The growth of collateral vessels is triggered by the pressure gradient between the coronary artery bed, led by the occlusion, and myocardial ischemia [35,36]. Myocardial ischemia stimulates CCC formation through the release of angiogenic growth factors such as transforming growth factor-α (TGF-α) and vascular endothelial growth factor (VEGF). However, the development or degree of CCC varies widely within patient groups. It is suggested that genetic factors and various other patient characteristics play a vital role in collateral vessel formation (age, diseases [hypertension, diabetes mellitus], and drug use [statins, b-blockers]) [37]. It has become widely known that inflammation plays a critical role in all stages of atherosclerosis. In addition to all variables previously enumerated in the development of CCC, studies indicate that inflammation and various inflammatory factors (e.g., NLR, PLR, and CRP) are associated with collateral formation [23,24,34].

Kalkan et al. [23] showed that NLR values are dramatically higher in stable angina pectoris patients with poor CCC. In addition, Açar et al. [24] confirmed the role of inflammation in CCC development based on their studies revealing that increased PLR levels are associated with poor collateral formation. Nevertheless, our findings did not support these results. In the multivariate analysis, we could not detect the association of NLR or PLR with poor CCC.

Kundi et al. [38] and Solorio et al. [9] found a negative correlation between CRP, which is a good inflammatory variable, and CCC formation. Although we found a relationship between CRP and poor CCC in the univariate analysis in our study, this relationship did not persist in the multivariate analysis.

Oxidant and antioxidant systems co-exist in a balanced way in organisms. Yet, oxidative stress occurs if this balance is disrupted in favor of oxidant substances. Free radicals formed due to increased oxidative stress react with membrane phospholipids in the cell membrane and facilitate the emergence of various cardiovascular diseases by playing a role in endothelial dysfunction and, thus, in the development of atherosclerosis [39]. Arai et al. [40] showed that heart failure after myocardial infarction is associated with antioxidant system suppression and increased oxidative stress. Zhao et al. [41] asserted that oxidative stress plays an important role in the pathogenesis and progression of ischemic–hypoxic encephalopathy. In their review, Sinha et al. reported that ROS play a remarkable role in the pathophysiology of hypertension [42]. Considering the balance between the oxidation and antioxidant system, it is not unwise to speculate that the antioxidant system is directly and/or indirectly instrumental in many disease groups.

Inflammation and oxidative stress damage many cells in the body. Experimentally elevated redox stress, as well as high oxidative stress, such as in metabolic syndrome and diabetes, inhibit the ability to develop CCC [43,44,45]. In experimental rat models, changes in the redox state were shown to be caused by the inhibition of collateral growth due to baseline oxidative stress [44,46].

Thiols are sulfur analogs of alcohols, and disulfides are structures containing neighboring double sulfur atoms [26]. It is thought that the thiol/disulfide balance plays an important role in the antioxidant mechanism. Thiols, which are non-enzymatic antioxidants, are among the main components of the mechanisms that protect against intracellular and extracellular damage. There are a few studies in the literature investigating the relationship between plasma thiol levels, the thiol/disulfide ratio and different forms of CAD. In one study, the disulfide/thiol ratio did not change significantly, but the decrease in the natural thiol levels was associated with the presence and severity of CAD [20]. In another study, the natural thiol, total thiol and disulphide levels in AMI patients were lower than in the control group, and the averages of the disulfide/natural thiol ratio and disulfide/total thiol ratio were higher in the AMI patients [47]. Altiparmak et al. [48] suggested that the total thiol is an independent diagnostic predictor of cardiac syndrome X. Also, Kundi et al. [49] and Kiziltunc et al. [21] reported that decreased native thiol and increased disulfide levels are associated with coronary artery ectasia and that a decreased native thiol/disulfide ratio is independently linked with a slow coronary flow. In our study, we found that sCAD patients with DM had poor CCC. This result was compatible with the data in the literature. In addition, we found an inverse relationship between poor CCC and the total thiol and disulfide levels, which were reported for the first time in the literature.

In our study, we found that the antioxidant level has a more effective determining role than the inflammation level in the formation of CCC in sCAD patients. Given the relationship between thiol/disulfide levels and CCC development, it is reasonable to point out some reasons that could explain this relationship. First off, in patients with stable CAD, the formation of CCC may depend on the ability of the antioxidant system to counteract oxidative stress and maintain the health of blood vessel walls. The lower levels of total thiol and disulfide in patients with poor CCC may indicate a compromised antioxidant defense system, making these patients more susceptible to oxidative damage. Moreover, when the antioxidant system is impaired, ROS levels may remain elevated, leading to increased inflammation and oxidative damage to blood vessels. This could potentially hinder the development of collateral vessels. Therefore, it can be speculated that the oxidative stress was higher in these patients, and it is reasonable to think that the thiol levels were further reduced to neutralize oxidative stress. Another mechanism is the important mediating role that VEGF plays in CCC development. The system called thioredoxin is a reductase that functions to re-convert disulfide bonds into thiol forms. There are studies showing that the and/or upregulation of this system is associated with increased VEGF and, thus, angiogenesis [50]. In contrast, patients with good CCC may have a more robust antioxidant system, enabling better protection against oxidative stress and inflammation, thereby facilitating collateral vessel formation. Indeed, in some CCC studies, it has been demonstrated that NLR is ineffective in predicting CCC. However, in the context of CCC prediction, it appears that NLR may not provide a comprehensive assessment of the inflammatory processes involved. It is possible that other inflammatory markers or pathways play a more significant role in CCC formation in patients with stable CAD; this could explain why NLR was found to be ineffective in some studies. Although we did not evaluate the levels of mediators, such as VEGF and TNF-α and thioredoxin, it is prudent to state that the decrease in the thiol level was associated with the increase in the thioredoxin levels and, therefore, with angiogenesis. The relationship between poor CCC and a low thiol level suggests that this system functions more. Although thiol/disulfide levels are generally thought to be inversely related, some studies show that their levels change in parallel [20,47]. The extracellular cysteine pairs in disulfides also protect reactive thiol groups, and their supportive effect on protein stability and function may be the reason for the low disulfide level [51].

The limitations of this study may include the small number of patients, the fact that it was a single-center study, and the fact that the parameters that may affect CCC were not measured, including mediators (VEGF, TGF α-β) and NO. In this article, the Rentrop classification was used to evaluate CCC. The patients included in this study were not subjected to intracoronary hemodynamic evaluation to measure CCC. Considering that intravascular hemodynamic evaluation is the gold standard for measuring CCC, this is perhaps the most important limitation of this study. In addition, we had no long-term follow-up information about the patients. Finally, the inclusion of former CAD patients may have affected the results.

## 5. Conclusions

The current study revealed that thiol/disulfide, an important member of the antioxidant system, is a predictor of the risk of poor CCC development in patients with sCAD. Our study is remarkable in that it compares the effects of the oxidant system and inflammatory markers on collateral formation. Although the two systems seem to work in parallel, we think that the oxidant system plays a more dominant role in collateral formation, as revealed by our study. Ultimately, more comprehensive and multi-center studies are needed to conduct better analyses of all possible predictors of poor CCC.

## Figures and Tables

**Table 1:** Demographic and clinical characteristics of the study populations [29].

Variables	Coronary Collateral Circulation		
	Poor (*n* = 49)	Good (*n* = 200)	*p* Value
Age (years)	67 (60–74)	62.5 (55–70)	0.015
Male gender (*n*, %)	37 (75.5)	144 (72)	0.621
DM (*n*, %)	20 (40.8)	42 (21)	0.004
HT (*n*, %)	27 (55.1)	105 (52.5)	0.744
Dyslipidemia (*n*, %)	17 (34.6)	71 (35.5)	0.916
Smoking (*n*, %)	25 (%51)	97 (49.5)	0.957
BMI (kg/m^2^)	27.4 ± 3.8	26.9 ± 2.9	0.639
Systolic blood pressure (mmHg)	126.8 ± 14,6	125.9 ± 16.3	0.721
Diastolic blood pressure (mmHg)	73.9 ± 18.5	74.6 ± 17.6	0.859
HR	77.3 ± 8.8	75.6 ± 9.2	0.231
LVEF (%)	55.4 ± 7.6	56.1 ± 9.1	0.551
Previous medications, *n* (%)			
Aspirin	14 (%28.6)	55 (%27.5)	0.912
ΒB	11 (%22.4)	46 (%23)	0.932
Angiotensin–aldosterone antagonists	9 (%18.3)	36 (%18)	0.923
Statin	11 (%22.4)	40 (%21)	0.813
Clopidogrel	3 (%6.1)	12 (%6)	0.894

BB: Beta blocker, BMI: body mass index, DM: diabetes mellitus, HR: heart rate, HT: hypertension, LVEF: left ventricular ejection fraction.

**Table 2:** Laboratory findings of the study populations [29].

Number of Patients	Coronary Collateral Circulation		
	Poor (*n* = 49)	Good (*n* = 200)	*p* Value
Glucose (mg/dL)	127.1 ± 85.2	109.2 ± 73.3	0.125
Creatinine (mg/dL)	0.91 ± 0.2	0.86 ± 0.2	0.153
ALT (U/L)	24.5 ± 11.2	27.2 ± 12.1	0.454
AST (U/L)	30.3 ± 8.8	35.5 ± 9.6	0.346
Total cholesterol (mg/dL)	189 (164–231)	186 (164–219)	0.701
High density lipoprotein cholesterol (mg/dL)	45 (35–53)	41 (35–47)	0.662
Low density lipoprotein cholesterol (mg/dL)	123 (87–145)	115 (93–137)	0.059
Triglyceride (mg/dL)	143 (106–204)	150 (105–215)	0.513
Hb (mg/dL)	14 (12.5–15)	14 (13–15)	0.816
PLT (10^3^/µL)	267 (232–323)	248 (209–291)	0.027
White blood cells (10^3^/µL)	9.3 (7.9–11.2)	8.9 (7.2–11.3)	0.331
hs-CRP (mg/L)	3.4 ± 2.9	2.6 ± 1.8	0.012
Neutrophil (10^3^/µL)	6.5 (5.5–8.5)	5.4 (3.4–6.4)	<0.001
Lymphocyte (10^3^/µL)	2 (1.5–3)	2 (2–3)	0.975
Neutrophil/Lymphocyte ratio (NLR)	3.2 (1.8–6.3)	2.7 (1.7–3.7)	0.008
Platelets/Lymphocyte ratio (PLR)	124 (97–186)	119 (94–116)	0.204
Total thiol (μmol/L)	278 (236–387)	432.2 (356.4–534.8)	<0.001
Native thiol (μmol/L)	258 (214–344)	384.6 (334.6–462.8)	<0.001
Disulfide (μmol/L)	16.2 (10.3–23.7)	22.3 (13.6–33.6)	0.003

ALT: alanine aminotransferase, AST: aspartate aminotransferase, Hb: hemoglobin, PLT: platelets, hs-CRP: high-sensitivity C-reactive protein, NLR: neutrophil/lymphocyte ratio, PLR: platelets/lymphocyte ratio.

**Table 3:** Angiographic data [29].

Rentrop Collateral Grades:	Coronary Collateral Circulation		
	Poor (*n* = 49)	Good (*n* = 200)	*p* Value
0	24		
1	25		
2		137	
3		63	
Position of chronic total occlusion			
Left anterior descending coronary artery	23 (%46.9)	98 (%49)	0.765
Left circumflex coronary artery	11 (%22.4)	45 (%22.5)	0.952
Right coronary artery	15 (%30.7)	57 (%28.5)	0.736
Number of diseased coronary artery			
One-vessel disease	14 (%28.6)	59 (%29.5)	0.811
Two-vessel disease	18 (%36.7)	68 (%34)	0.797
Three-vessel disease	17 (%34.7)	73 (%36,5)	0.812
SYNTAX score	19.7 ± 4.5	20.3 ± 5.3	0.645

**Table 4:** Univariate and multivariate predictors of well-developed coronary collateral circulation in patients with stable coronary artery disease [29].

	Univariate Analysis			Multivariate Analysis		
	Odds Ratio	95% CI	*p* Value	Odds Ratio	95% CI	*p* Value
DM	2.594	1.336–5.038	0.005	2.671	1.238–5.761	0.012
Age	1.039	1.007–1.073	0.018			
PLT	1.003	0.999–1.000	0.097			
Hs CRP	1.182	1.033–1.351	0.015			
NLR	1.081	1.002–1.166	0.045			
Total Thyol	1.011	1.007–1.015	<0.001	1.012	1.008–1.017	<0.001
Disulfide	0.961	0.935–0.989	0.006	1.022	1.000–1.044	0.044

DM: diabetes mellitus, NLR: neutrophil/lymphocyte ratio, CRP: C-reactive protein, PLT: platelet.

## Data Availability

Not available.
